# Supplementary material for: Ozone as an environmental driver of influenza
Source: Nat Commun. 2024 May 4;15:3763. doi: 10.1038/s41467-024-48199-z (PMC11069565; doi:10.1038/s41467-024-48199-z)
Supplement: Supplementary file 1 — Supplementary Information [file 41467_2024_48199_MOESM1_ESM.pdf]

Supplementary Information

Ozone as an environmental driver of influenza

Author Information

Fang Guo[1], Pei Zhang[1], Vivian Do[2], Jakob Runge[3,4], Kun Zhang[5,6], Zheshen Han[1], Shenxi Deng[1], Hongli Lin[1], Sheikh Taslim Ali[1,7], Ruchong Chen[8], Yuming Guo[9], Linwei Tian[1,10*]

[1] *School of Public Health, The University of Hong Kong, Pok Fu Lam, HKSAR, PR China*

[2] *Mailman School of Public Health, Columbia University, New York, USA*

[3] *Deutsches Zentrum für Luft- und Raumfahrt (DLR), Institut für Datenwissenschaften, Jena, Germany*

[4] *Technische Universität Berlin, Berlin, Germany*

[5] *Department of Philosophy, Carnegie Mellon University, Pittsburgh, Pennsylvania, USA*

[6] *Machine Learning Department, Mohamed bin Zayed University of Artificial Intelligence, Abu Dhabi, UAE*

[7] *Laboratory of Data Discovery for Health Limited, Hong Kong Science Park, New Territories, HKSAR, PR China*

[8] *State Key Laboratory of Respiratory Disease, National Clinical Research Center for Respiratory Disease, National Center for Respiratory Medicine, Guangzhou Institute of Respiratory Health, Department of Allergy and Clinical Immunology, The First Affiliated Hospital of Guangzhou Medical University, Guangzhou, PR China*

[9] *Climate, Air Quality Research Unit, School of Public Health and Preventive Medicine, Monash University, Melbourne, Australia*

[10] *Institute for Climate and Carbon Neutrality, The University of Hong Kong, Pok Fu Lam, HKSAR, PR China*

These authors contributed equally: Fang Guo, Pei Zhang, Vivian Do

[*]Corresponding author: Dr. Linwei Tian

Address: School of Public Health, The University of Hong Kong, 7 Sassoon Road, Pokfulam, Hong Kong.

Telephone: +852 39176351

E-mail: linweit@hku.hk

Supplementary Results to Main Manuscript

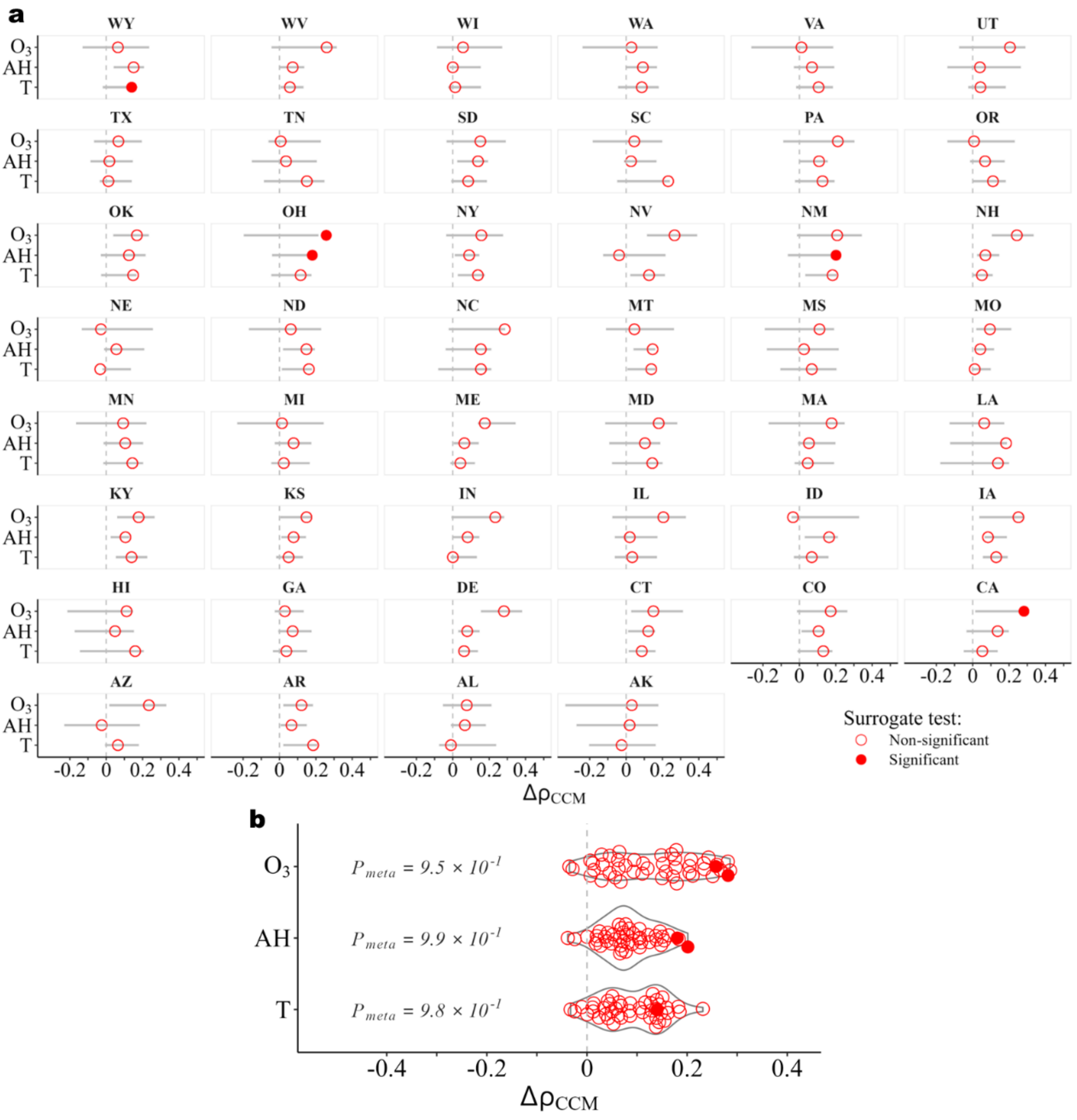

Figure S1. Reverse causality test for the effect of influenza intensity, at 1-week lag, on environmental measurements (ozone [O_3], absolute humidity [AH], temperature [T]) by convergent cross-mapping (CCM). a State-specific observed CCM skills (as circles), $\Delta\rho_{CCM}$, and their null distribution in 1,000 seasonal surrogates (as line ranges). Circles are filled to signify the measured $\Delta\rho_{CCM}$ for each state exceeding 95% of its null values. **b** Summary of state-specific $\Delta\rho_{CCM}$ values in violin plots. When summing the logs of state-specific P values to obtain the meta-significance estimate for the nation (P_{meta}), none of the 3 environmental factors are found to be driven by influenza at a significance threshold of $P_{meta} < 1.0 \times 10^{-3}$.

Table S1. Summary of assumptions and properties of the three causal inference methods

	**CCM** [1]	**PCMCI+** [2,3]	**GLM** [4]
Oriented System	Deterministic	Stochastic	Stochastic
Theoretical Framework	State space reconstruction for dynamical system	Graphical modelling in Bayesian networks	Regression modelling
Test of causation	Coupling in the dynamical system	Conditional independence	Statistical association in parametric model
Assumptions	Low-dimensional deterministic dynamics with limited stochasticity	Causal sufficiency;<br>Causal Markov Condition;<br>Faithfulness;<br>Causal stationarity	Appropriate link function and variance function;<br>Independently distributed residuals

Abbreviations: CCM, convergent cross mapping; PCMCI+, Peter-Clark-momentary-conditional-independence plus; GLM, Generalised Linear Model

Terminology:

- Deterministic: to describe a dynamical system of which the future states depend on the initial condition and evolve following certain equation without randomness.
- Stochastic: to describe a system composed of random variables which can be studied by mathematical theory of probability.
- State space reconstruction: reconstructing the state space of original dynamical system from a single time series with delayed coordinates.
- Graphical modelling: a framework combining the rigor of Bayesian probabilistic networks to analyse and represent complex relationships between variables in a system.
- Causal sufficiency: All confounders of the relevant variables are observed in the given dataset.
- Causal Markov Condition: every variable, given its direct causes observed or controlled for, is independent of its non-effects (i.e., variables that are not causally influenced by it).
- Faithfulness: The causal graph represents exactly the statistical independencies in the distribution implied by d-separation.
- Causal stationarity: the existence/absence of links in the causal graph hold over time, the strength of which could yet vary.

Table S2. Determination of key parameters in state-wise Empirical Dynamic Modelling (EDM) analysis

State	E	θ	State	E	θ
**AK**	2	0.01	**MS**	2	0.01
**AL**	3	0.5	**MT**	4	0.1
**AR**	5	3.0	**NC**	3	0.3
**AZ**	3	0.01	**ND**	4	0.01
**CA**	6	0.01	**NE**	3	0.3
**CO**	5	0.75	**NH**	6	0.5
**CT**	2	0.75	**NM**	4	0.3
**DE**	5	2.0	**NV**	3	0.5
**GA**	3	3.0	**NY**	5	0.75
**HI**	6	2.0	**OH**	6	0.01
**IA**	6	0.1	**OK**	6	1.5
**ID**	2	0.01	**OR**	2	0.1
**IL**	4	1.5	**PA**	5	0.3
**IN**	5	0.3	**SC**	3	1.0
**KS**	6	1.5	**SD**	2	3.0
**KY**	5	0.3	**TN**	4	0.1
**LA**	3	0.75	**TX**	6	0.01
**MA**	4	0.3	**UT**	6	0.5
**MD**	2	0.01	**VA**	4	0.01
**ME**	5	0.01	**WA**	4	0.75
**MI**	4	0.5	**WI**	6	0.01
**MN**	6	0.1	**WV**	6	1.0
**MO**	5	0.01	**WY**	5	0.01

Notes:

- E is the embedding dimension for state space reconstruction. E was chosen state-wise over [2,6] where the maximum of predictability is achieved in a data-driven manner. Here, the upper limit of E=6 was specified because the maximum E that we can recover with a time series of consecutive 35 or 34 data points per year scales roughly as its square root [5].
- θ, controlling degree of nonlinearity, was optimized over the range [0.01, 9] that maximizes the univariate S-map forecast performance using leave-one-out cross-validation over the whole time series.

Supplementary Analyses: multivariate forecast improvement (MFI)

In the main manuscript, we used convergent cross mapping (CCM) analysis under the empirical dynamic modelling (EDM) framework to test causal relationships in a bivariate manner. Here, MFI analysis which lies in multivariate state-space reconstruction (SSR) was used additionally to test whether and how well the hypothesized driving factor could forecast the influenza dynamics [6-8]. The concept of MFI test is that: considering two time series Xt and Yt, if variable X causes variable Y, then better forecast of Y should be obtained by incorporating the information from X and Y simultaneously (i.e., multivariate model) rather than by using the information from Y only (i.e., univariate model). Here, the forecast skill is measured by correlation coefficient (ρ) between the predicted and observed values of Yt time series. And the improvement in forecast skill is quantified by calculating $\Delta\rho_{MFI} = \rho_{multi} - \rho_{uni}$, that is, the difference between the forecast skills from multivariate and univariate SSR models.

To compare how well the putative drivers alone or in combination could better predict the influenza intensity, we used nonparametric method to examine whether the overall distribution of $\Delta\rho_{MFI}$ from each state is greater than 0 and also different from each other. One-sample Wilcoxon test and paired two-sample Wilcoxon test were used as appropriate respectively. Similar to CCM analysis reported in the main text, we also supplemented seasonal surrogates' significance test for MFI analysis on a state-by-state basis. The $\Delta\rho_{MFI}$ obtained for the real time series was compared against its null distribution built by 1000 surrogates with randomized seasonal anomalies. A P-value was computed for rejecting the null hypothesis of shared seasonality.

The results of MFI analysis largely coincide with the main-text CCM results, providing further confidence that ambient O_3 is a likely more direct environmental driver of influenza dynamics in the USA. As shown in **Figure S2**, when each of the three environmental variables at 1-week lag is separately included in the EDM, O_3 but not AH and T could significantly improve the forecast skill for influenza ($P < 2.9 \times 10^{-5}$ for O_3; $P > 5.6 \times 10^{-1}$ for AH; $P > 2.5 \times 10^{-2}$ for T). Wilcoxon tests further indicate that adding O_3 as an embedding coordinate could lead to more forecast improvement of influenza than adding either AH ($P < 3.6 \times 10^{-6}$) or T ($P < 4.5 \times 10^{-4}$). The forecast improvement by AH or T alone can be further enhanced by the addition of O_3 as another embedding ($P < 1.1 \times 10^{-6}$and $P < 1.1 \times 10^{-3}$, respectively). When state-by-state observed MFI levels were compared with the expected values of seasonal surrogates, nation-wide meta-significance test by Fisher's method showed that only O_3 could improve the forecast of influenza intensity at lag 1 ($P_{meta} < 2.6 \times 10^{-2}$), but neither AH ($P_{meta} > 9.4 \times 10^{-1}$) nor T ($P_{meta} > 2.4 \times 10^{-1}$) (**Figure S3**).

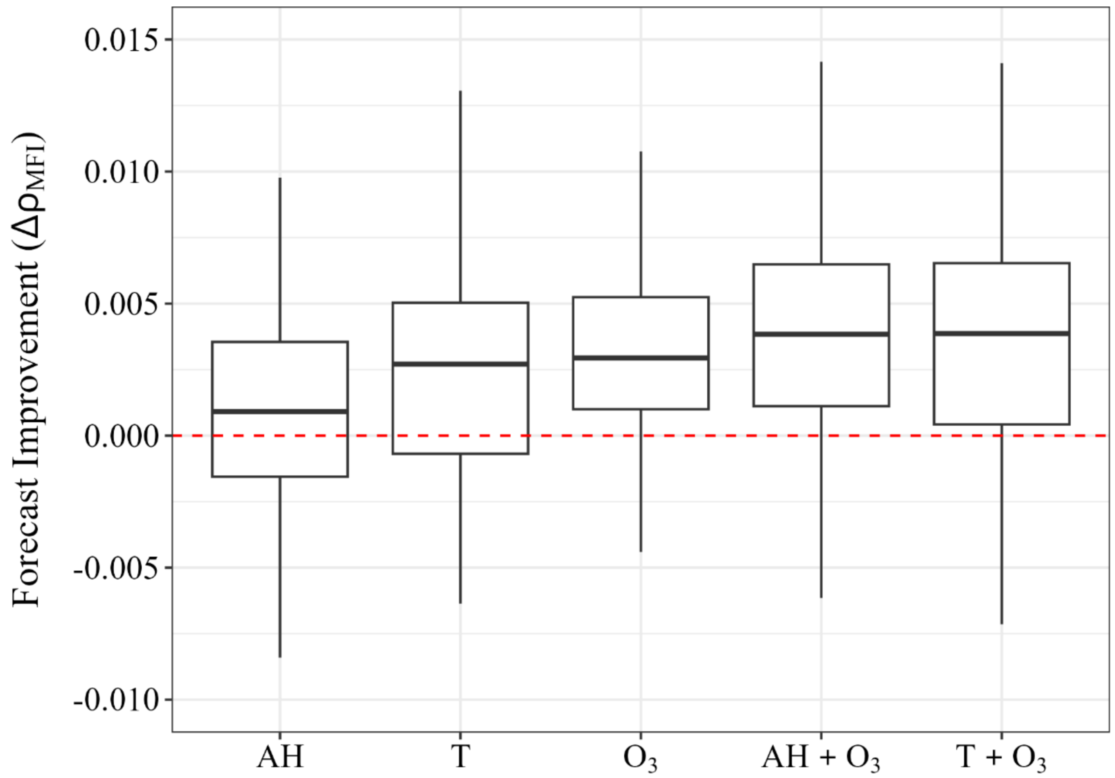

Figure S2. State-combined summary of multivariate forecast improvement (MFI) on influenza intensity by putative environmental drivers (ozone [O_3], absolute humidity [AH], temperature [T]) at 1-week lag. The forecast improvement (denoted as $\Delta\rho_{MFI}$) indicates the improvement in forecast skill (ρ) by adding embedding coordinates of driving variable(s) in predicting the response variable. Each boxplot is a summary of the state-specific observed $\Delta\rho_{MFI}$ values obtained in different MFI models. The middle line represents the median, the box covers the interquartile range (IQR), and the whiskers extend from the hinge to the largest/smallest value at most 1.5×IQR. Outliers are not shown.

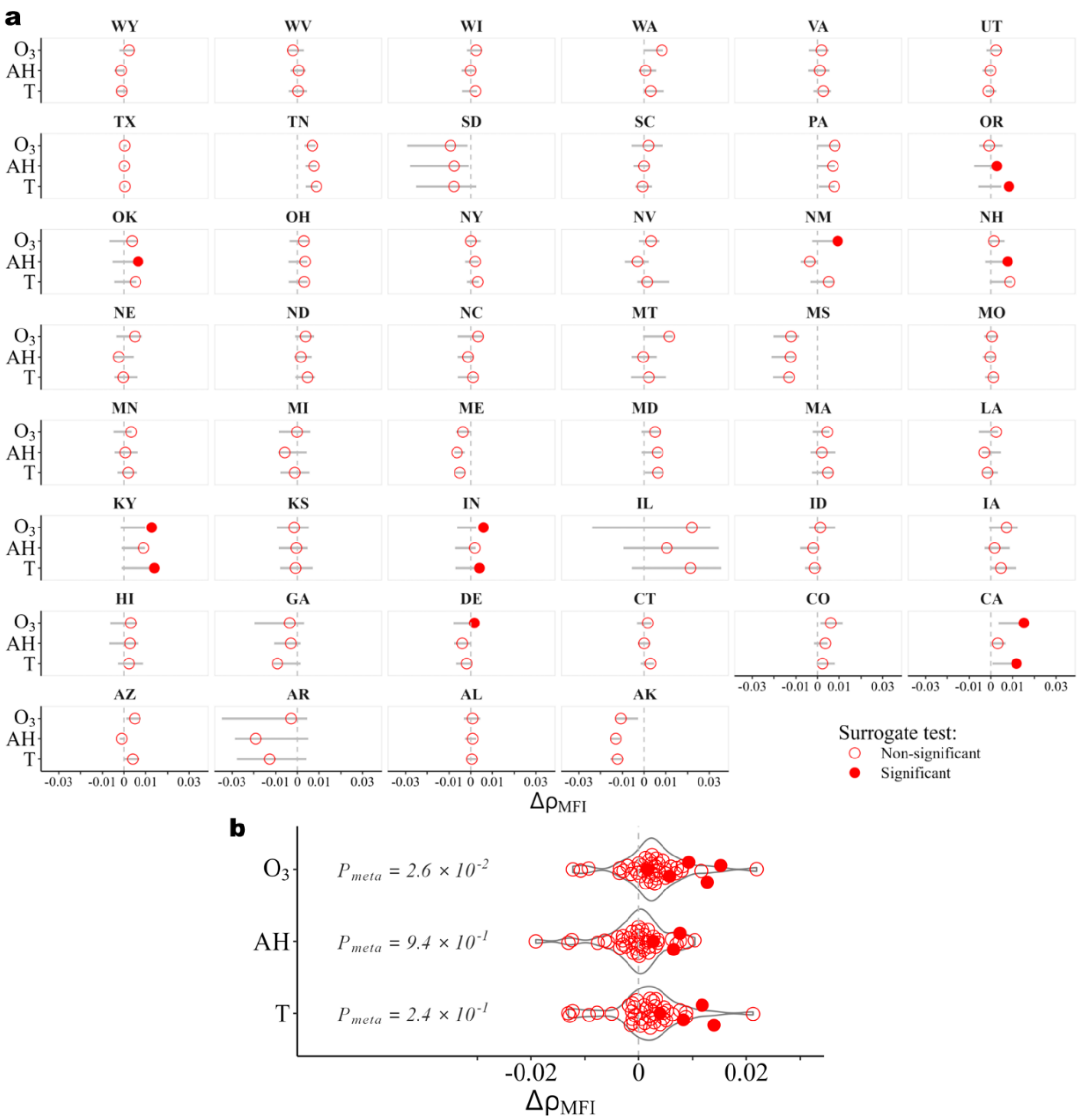

Figure S3. Multivariate forecast improvement (MFI) on influenza activity by putative environmental drivers (ozone [O_3], absolute humidity [AH], temperature [T]) at 1-week lag in the states of USA. a State-specific observed MFI skills (as circles), $\Delta\rho_{CCM}$, and their null distribution in 1,000 seasonal surrogates (as line ranges). Circles are filled to signify the measured $\Delta\rho_{CCM}$ for each state exceeding 95% of its null values. **b** Summary of state-specific $\Delta\rho_{MFI}$ values in violin plots. Meta-significance estimate for the nation (P_{meta}) is tested by summing the logs of state-level P values; MFI causality is deemed significant with $P_{meta} < 1.0 \times 10^{-3}$.

Supplementary Analyses: fixed effects linear model (FELM)

In the main manuscript, we used classic time series regression method, generalised linear model (GLM) with a quasi-Binomial link (i.e., logit function) to estimate the statistical association of O_3, AH, and T with influenza intensity at each state, and then pooled the state-level coefficients via meta-analysis. Here, as a robustness check, we used another regression-based method, FELM, by including “state” as fixed effects during nation-wide analysis, to control for time-invariant factors specific to each state (e.g., socioeconomic or policy differences) that may influence the baseline influenza intensity [9]. Calendar year was included as fixed effects in the model to control for time-varying unobserved factors (e.g., potential reporting issues that may trend over time). Then, the core FELM was re-run by further controlling for seasonality and school semesters/holidays (as dummy variables) that may bias the link of interest on a seasonal scale. To account for strong autocorrelation caused by disease transmission, we took the logarithm of 1-week and 2-week lagged outcome variables (i.e., $log(Y_{t-1})$ and $log(Y_{t-2})$) as covariates in the model. When estimating the relationship of O_3 with influenza, the same-week AH and T are simultaneously included in the model as a linear term to control for potential confounding. The “lfe” (version 2.9.0) [10] was adopted to fit the fixed-effects linear regression model.

Table S3 summarizes the lag-specific statistical relationships between environmental factors and influenza activity from the FEML regressions, by contract with those from GLM regressions. Ambient O_3 was found to reduce influenza activity ($P < 1.0 \times 10^{-3}$) at lag 1 (week), consistently by the two regression-based methods. While GLM revealed a negative link between AH and influenza at lag 1, FELM detected none. The negative association of 2-week lagged air T with influenza, though presented simultaneously by FELM and GLM regressions, is not supported by CCM and PCMCI+ analyses shown in the main manuscript (**Table 1**). **Figure S4** further visualizes the state-wise regression results by FELM at the lag 1. Panel **a** showed that the 1-week lagged statistical associations of O_3 with influenza intensity are generally negative at the state level. By conducting nationwide analysis with state included as fixed effects (panel **b**), one SD increment in O_3 concentration is associated with a reduction of 0.106 (CI: -0.163, -0.048; $P < 1.4 \times 10^{-9}$) in logit-transformed influenza intensity one week after.

Table S3. Effects of environmental factors on influenza activity estimated by two regression-based methods, based on weekly state-level data of the USA during 2010-2015

	GLM		FELM	
	Effect	*P*-value	Effect	*P*-value
**Ozone**				
Lag 0	-0.016	5.4×10^{-1}	**-0.113**	$\mathbf{6.4\times10^{-10}}$
Lag 1	**-0.102**	$\mathbf{5.9\times10^{-5}}$	**-0.106**	$\mathbf{1.4\times10^{-9}}$
Lag 2	-0.017	3.9×10^{-1}	**-0.087**	$\mathbf{4.3\times10^{-7}}$
**Absolute humidity**				
Lag 0	-0.037	4.3×10^{-1}	-0.083	8.5×10^{-3}
Lag 1	**-0.310**	$\mathbf{6.7\times10^{-8}}$	-0.076	1.3×10^{-2}
Lag 2	-0.020	6.6×10^{-1}	-0.018	5.6×10^{-1}
**Temperature**				
Lag 0	0.075	2.6×10^{-2}	**-0.195**	$\mathbf{1.5\times10^{-8}}$
Lag 1	0.076	1.1×10^{-1}	**-0.256**	$\mathbf{2.8\times10^{-14}}$
Lag 2	**-0.158**	$\mathbf{2.0\times10^{-6}}$	**-0.257**	$\mathbf{1.7\times10^{-14}}$

Abbreviations: GLM, generalised linear model; FELM, fixed effects linear model.

Note: In the 2 sets of results, bold values suggest statistically significant relationships with statistical significance of $P < 1.0 \times 10^{-3}$ (two-sided). With GLM, regressions were first performed at state-level and then pooled by meta-analysis; while with FELM, state-level data were pooled to run nation-wide regression but with fixed effects defined by "state".

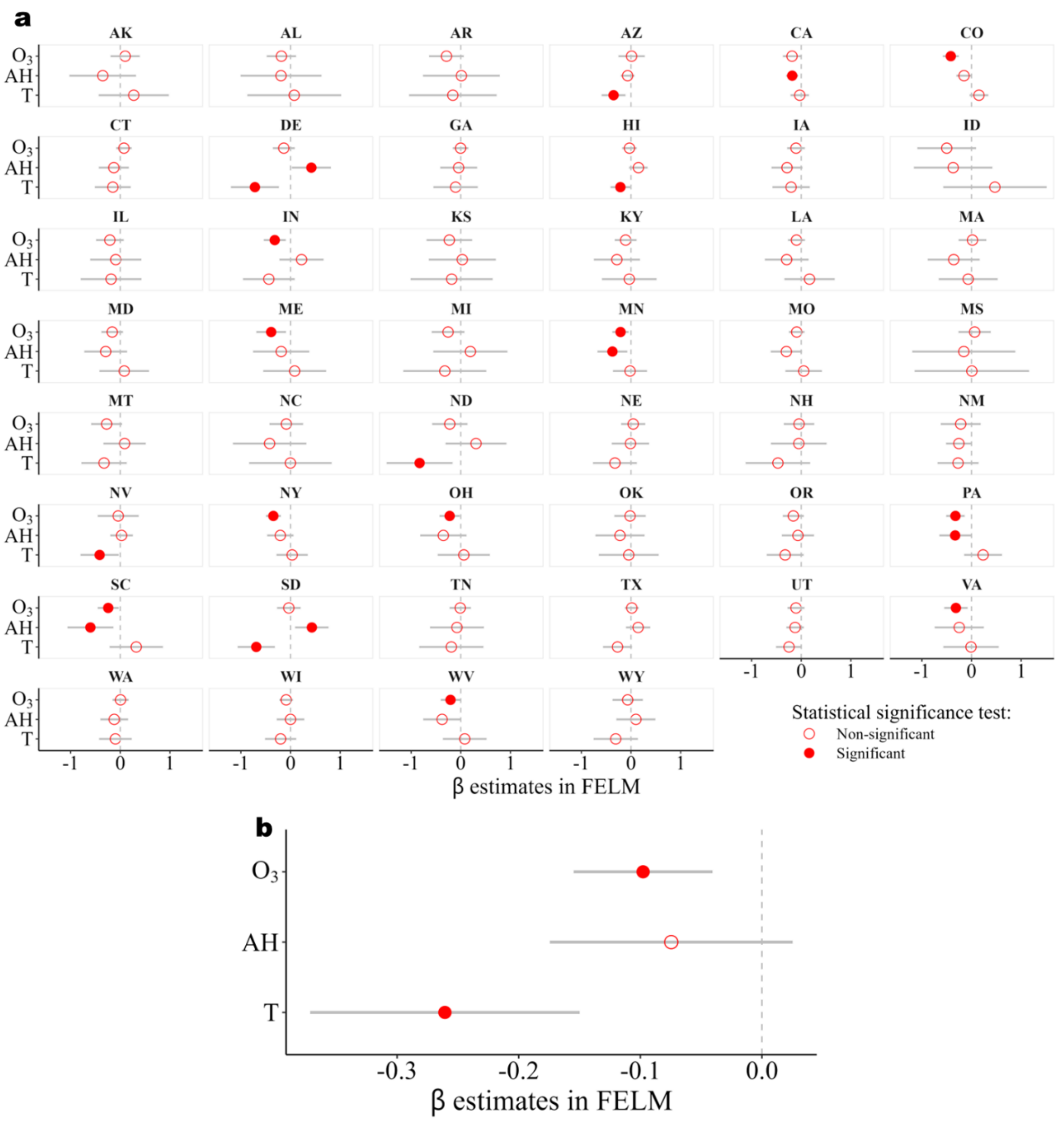

Figure S4. Effects of environmental factors on influenza intensity, at 1-week lag, estimated by Fixed Effects Linear Model (FELM). a State-level point estimates of regression coefficients (β) and their 95% confidence intervals shown as circles and line ranges, respectively; Circles are filled when the *P*-value for statistical significance test is <0.05 (two-sided). **b** Nationwide point risk estimates with the corresponding 99.9% confidence intervals shown as circles and line ranges, respectively, by including “state” fixed effects. Circles are filled when the *P*-value is <0.001 (two-sided) during nationwide analysis. O_3: ozone; AH: absolute humidity; T: temperature.

Step-by-step Demonstration of Data Analyses

Sketch

This study, ***Ozone as an environmental driver of influenza***, analyzed a total of 46 states to investigate the environmental drivers of influenza intensity in the USA. The analytical framework consists of three distinct but complementary methods: convergent cross mapping (CCM), Peter-Clark-momentary-conditional-independence plus (PCMCI+), and generalized linear model (GLM), to strive for detection of causal links notably by evidence accumulation.

Here, to facilitate open science, we generate the current file as supporting information for a streamlined and intuitive demonstration of the major steps involved in data analysis. The entire data set and analysis codes are accessible at https://zenodo.org/records/10892898.

1. CCM

We firstly used the data-driven approach, CCM, which is oriented for causal discovery in dynamical systems, to explore the potential driving role of ambient ozone (O_3), absolute humidity (AH), and temperature (T) in influenza dynamics of the USA during 2010-2015.

To maximize computational efficiency, the R script of CCM analyses is set up to leverage parallel computing capabilities. With data loaded, 'fluP' column of proportion type is a proxy measure of influenza intensity in the community. For this reason, employing a quasi-binomial link with a *logit* function (that is, $log\left(\frac{Y}{1-Y}\right)$) is often considered appropriate in the GLM regressions. Drawing parallels with GLM, the same logit-transformed 'fluP' was also used in both CCM and PCMCI+ analyses. This transformation provides a consistent framework for interpreting the results across different analytical methods. Lastly, the data undergoes normalization, where each variable in the data set is scaled to have a mean of zero and a standard deviation of one. This process can be essential for many statistical techniques as it puts variables on a common scale.

```
###### Load packages, data, and functions
# packages
packages=c('tidyverse','knitr','lubridate','rEDM','metap',
           'doParallel','foreach','imputeTS', "kableExtra",
           'ggplot2', 'ggpubr', 'ggthemes', 'cowplot',
           'customLayout', 'patchwork', 'grid', 'gridExtra',
           'usmap', 'maps', 'metap', 'scales', 'ggridges',
           'ggforce', 'ggbeeswarm')
lapply(packages, require, character.only=T)

# parallel computing parameters
cores_all=detectCores()
cores=ifelse(cores_all<9,4,cores_all-2)
core_type='PSOCK' # parallel backend

num_sample=100
num_surr=1000

## load data and functions
```

```
load("Data_fluseason.rda")
df <- usa_Flu_P_proxy_Data_B
df$date <- as.Date(df$date )

## logit transformation of fluP
dfA=df
logitTransform <- function(p) { log(p/(1-p)) }
dfA$logitfluP <- logitTransform(dfA$fluP)
dfA$fluP <- dfA$logitfluP

## normalization of variables
normFunc=function(x){(x-mean(x, na.rm = T))/sd(x, na.rm = T)}

df_smapc=dfA %>% group_by(state) %>%
  mutate_at(3:ncol(.),normFunc) %>% ungroup()
```

1.1 Seasonality and surrogate data

To rule out the possibility of spurious causal signal due to shared seasonal forcing, a null distribution of cross-mapping skill using 1000 seasonal surrogate time series of environmental variables is formed to compare with that obtained using the original data. Surrogate data are produced by `rEDM::SurrogateData` function where `method="seasonal"` is set.

Calculate parameter `spar` for smoothing splines

To generate seasonal surrogate data, the first step is calculating parameter `spar`, which controls the trade-off between fidelity to the data and roughness of the function estimate in smoothing spline, indicative of the underlying seasonality pattern.

```
# whole-year environmental data
load("Data_wholeyear.rda")
df <- usa_Flu_P_proxy_Data_full_B
df$date <- as.Date(df$date)

# normalization of variables
dfB=df
dfB_smapc=dfB %>% group_by(state) %>%
  mutate_at(3:ncol(.),normFunc) %>% ungroup()

#select variables
df_flu=dfB_smapc %>% select(date,state,ah,o3,temp) %>%
  group_by(state) %>% gather(key,value,-c(date,state))

df_flu <- dfB_smapc %>%
  select(date, state, ah, o3, temp) %>%
  mutate_at(vars(ah, o3, temp), ~{attributes(.) <- NULL; .}) %>%
  group_by(state) %>%
  gather(key, value, -c(date, state))

# calculate spar values for independent variables
fn_state_spar=function(states,keys){

  splineres <- function(spar){
    res <- rep(0, length(x))
```

```
    for (i in 1:length(x)){
      mod <- smooth.spline(x[-i], y[-i], spar = spar)
      res[i] <- predict(mod, x[i])$y - y[i]
    }
    return(sum(res^2))
  }

  x=df_flu %>% ungroup() %>%
    filter(state==states & key==keys) %>%
    select(date) %>% pull() %>% as.numeric()

  y=df_flu %>% ungroup() %>%
    filter(state==states & key==keys) %>%
    select(value)  %>% pull() %>% as.numeric()

  spars <- seq(0, 1.5, by = 0.1)
  ss <- rep(0, length(spars))

  ss=foreach(i = 1:length(spars),
             # .packages = c("rEDM","tidyverse"),
             # .export=c('num_sample','num_surr'),
             .combine=rbind,
             .inorder=FALSE)  %dopar% {
               targetCol = paste("T", i, sep = "")
               ss[i] <- splineres(spars[i])
             }

  spar=spars[which.min(ss)]
  data.frame(state=states,plt=keys,spar)
}


plist=list(states=unique(df_flu$state),
           keys=c('o3','ah','temp')) %>% cross_df()

cl <- makeCluster(cores[1],type = core_type)
registerDoParallel(cl)
flu_spar=plist %>% pmap_df(fn_state_spar)
stopCluster(cl)
```

Calculate parameter* `alpha` *for surrogate data

After obtaining *`spar`*, we bring it into smoothing splines to get day-of-year anomalies for environmental variables. Then we calculate the standard deviation (SD) of these anomalies to get the parameter *`alpha`*, which is the additive noise factor to produce surrogate data.

```
# Calculate SD values of anomalies for environmental variables (that
 is, `alpha` to be used in the `rEDM::SurrogateData` function)

yearday_anom <- function(t,x,spars){
  # t: date formatted with POSIXt
  # x: time-series values to compute seasonal mean and anomaly
  doy <- as.numeric(strftime(t, format = "%j"))
  I_use <- which(!is.na(x))
```

```
  # create time indices to use for smoothing, replicating data to "w
rap around"
  doy_sm <- rep(doy[I_use],3) + rep(c(-366,0,366),each=length(I_use)
)
  x_sm <- rep(x[I_use],3)
  xsp <- smooth.spline(doy_sm, y = x_sm, w = NULL,
                       spar = spars, cv = NA,
                       all.knots = TRUE,keep.data = TRUE, df.offset
= 0)
  xbar <- data.frame(t=t,doy=doy) %>%
    left_join(data.frame(doy=xsp$x,xbar=xsp$y),by='doy') %>%
    select(xbar)
  out = data.frame(t=t,mean=xbar,anomaly=(x - xbar))
  names(out) <- c('date','mean','anomaly')
  return(out)
}

fn_anomaly_PNAS=function(states,plts){
  vec_t=df_flu %>%
    ungroup() %>%
    filter(state==states & key==plts) %>%
    select(date) %>% pull()

  vec_x=df_flu %>%
    ungroup() %>%
    filter(state==states & key==plts) %>%
    select(value) %>% pull()

  spars=flu_spar %>% filter(state==states & plt==plts) %>%
    select(spar) %>% pull()

  df_9=yearday_anom(vec_t,vec_x,spars)
  sd=sd(df_9$anomaly,na.rm=TRUE)
  data.frame(states,plt=plts,spar=spars,sd_PNAS=sd)
}

plist=list(states=unique(df_flu$state),
           plts=c('o3','ah','temp')) %>% cross_df()

sd_data=plist %>% pmap_df(fn_anomaly_PNAS)
```

Produce seasonal surrogate data

Then we employ `rEDM::SurrogateData` to produce surrogate data. The codes are presented below, which are used in function `fn_season_ccm` later.

```
fn_surr_data=function(data,ST,plts, tp_value){
  df=data %>%
    filter(state==ST) %>%
    mutate(plt=lag(.data[[plts]],-tp_value)) %>%
    filter(!(is.na(plt))) %>%
    select(date,"plt")

  alpha=sd_data %>% filter(states==ST & plt==plts) %>%
    select(sd_PNAS) %>% pull()
```

```
  set.seed(2019)
  surr_data=
    SurrogateData(unlist(df[,"plt"]), method = "seasonal",
                  T_period = 52.18,
                  num_surr = num_surr,
                  alpha=alpha) %>%
    as.data.frame()
  df=df %>% select(-"plt")
  surrA=bind_cols(df,surr_data)
}
```

1.2 Determine the key parameters

Determine optimal E

Since our analysis is based on flu-season data (from October throughout May), we mark the time indexes to be used for analysis. The function `jishu` firstly determines whether a number is even or odd. Subsequent to this, the `make_pred_nozeroL` function is designed to process a data set, adding in pertinent time indexes and extracting specific date-related data points, namely the first day of October for each year and the last day of May in the next year. The essence of this function is to form a structured sequence of data points, facilitating the downstream analysis limited to flu seasons.

The third function, `fn_E_smapc`, dives deeper into the data, filtering it based on specific states and omitting any incomplete entries. This function harnesses the previously defined `make_pred_nozeroL` function to delineate specific ranges within the data set. With these established, it calculates an embedding dimension (E) for the data set — a key parameter for reconstructing a properly embedded state space, laying the foundation for CCM analysis. The value of E was chosen over the range of 2 to 6 where the maximum of univariate predictability is achieved via leave-one-out cross-validation. The lower limit of E=2 was specified in order to embed at least one external variable to reconstruct multivariate manifold; the upper limit of E=6 was specified because the maximum E should not be larger than the square root of the consecutive time series data length, which was 35 or 34 weekly data points in influenza season in the current study.

```
###### Determine optimal E for the system by each state
jishu <- function(x){
  ifelse(x%%2 ==0,F,T)
}

make_pred_nozeroL <- function(dat){
  dat <- dat %>% mutate(year=year(date),month=month(date),day=day(da
te),n=1:nrow(dat))
  dat1 <- dat %>% filter(month==5) %>% group_by(year) %>% filter(day
==max(day))
  dat2 <- dat %>% filter(month==10) %>% group_by(year) %>% filter(da
y==min(day))
  I_zero_strings <- c(dat1$n,dat2$n)[order(c(dat1$n,dat2$n))]

  if(I_zero_strings[1]!=1 | jishu(length(I_zero_strings))==T) {
    I_zero_strings=c(1,I_zero_strings)
```

```
  } else {
    I_zero_strings=I_zero_strings
  }

  if (I_zero_strings[2]==1) {
    I_zero_strings=I_zero_strings[3:length(I_zero_strings)]
  }

  lib_out <- matrix(I_zero_strings,ncol=2,byrow=T)
  return(lib_out)
}

fn_E_smapc=function(data,ST,y){

  df=data %>%
    filter(state==ST) %>%
    select(date,y) %>%
    na.omit()

  M <- nrow(df)

  lib <- make_pred_nozeroL(df)
  pred <- make_pred_nozeroL(df)

  E=EmbedDimension(dataFrame=df,
                   lib=lib,
                   pred=pred,
                   columns=y, target=y,
                   maxE=6,
                   showPlot=F)
  temp=data.frame(dis=y,E,ST)
  temp}

plist=list(data=list(df_smapc),y=c('fluP'),ST=unique(dfA$state)) %>%
  cross_df()

E_smapc_out=plist %>% pmap_df(fn_E_smapc)

E_smapc =E_smapc_out %>% filter(E %in% 2:6) %>%
  group_by(dis,ST) %>%
  filter(rho==max(rho))  %>%
  as.data.frame()
```

Determine optimal theta

The provided code is aimed at determining the optimal value of a parameter, theta (θ), in the context of nonlinear time series prediction (that is, S-map [sequential locally weighted global linear map]). This theta parameter represents the degree of nonlinearity in the system, and its optimal value can be critical for generating accurate forecasts.

```
###### Determine optimal theta for S-map by each state
fn_theta_justY=function(data, ST, dis, theta){

  E=E_smapc[E_smapc[,'ST']==ST & E_smapc[,'dis']==dis,'E']
```

```
  df=data %>%
    filter(state==ST) %>%
    select(date,dis)

  M <- nrow(df)

  lib=make_pred_nozeroL(df)
  pred=make_pred_nozeroL(df)

  rho_theta = PredictNonlinear(dataFrame = df,
                               embedded = FALSE,
                               columns = dis,
                               target = dis,
                               Tp=1,
                               theta=theta,
                               lib=lib,
                               pred=pred,
                               showPlot = FALSE,
                               E = E)

  best_theta_df=rho_theta %>%
    mutate(dis=dis, state=ST, theta=theta)
}

plist=list(data=list(df_smapc),ST=unique(dfA$state),
           dis=c('fluP'),
           theta=c(0.01, 0.1, 0.3, 0.5, 0.75, 1, 1.5, 2, 3, 4,
                   5, 6, 7, 8, 9)) %>% cross_df()

cl <- makeCluster(cores[1],type = core_type)
registerDoParallel(cl)
theta_out=foreach(i = 1:nrow(plist),
                  .packages = c("rEDM","tidyverse",'lubridate'),
                  .combine=rbind,
                  .inorder=FALSE)  %dopar% {
                    theta_out=plist[i,] %>% pmap_df(fn_theta_justY)
                  }
stopCluster(cl)

best_theta=theta_out %>%
  group_by(state) %>%
  filter(rho==max(rho)) %>%
  as.data.frame()
```

1.3 CCM causality test

CCM is rooted in *Takens' Theorem* and serves as a pivotal tool for causality testing within the Empirical Dynamic Modeling (EDM) framework, from a dynamical systems perspective. Essentially, the core premise is that if variable X causally impacts variable Y, then a sufficiently delayed embedding of the driven time series Y_t (i.e., its reconstructed manifold M_Y) should encapsulate the requisite dynamic information to cross-predict the present values of X_t. Conversely, the opposite prediction, from X to Y, is not necessarily valid. With optimal E obtained, we can

calculate CCM for real and surrogate data. Here we combine original data with surrogates first, and then conduct CCM for them together.

```
###### Surrogate CCM
fn_season_ccm=function(data,ST,x,y,tp_value){

  df=data %>%
    filter(state==ST) %>%
    select(date,y,x)

  E=E_smapc[E_smapc[,'ST']==ST & E_smapc[,'dis']==y,'E']

  alpha=sd_data %>% filter(states==ST & plt==x) %>%
    select(sd_PNAS) %>% pull()

  surr_data <- fn_surr_data(dfB_smapc,ST,x,0)

  all_data <- df %>% left_join(surr_data,by="date")

  names(all_data) = c("date", y, 'T1',paste0("T", 2:(num_surr+1)))

  m=nrow(all_data) %>% as.data.frame()

  libSize =c(E+2,m-E-2)

  rho_surr <- NULL

  for (i in 1:(num_surr+1)) {
    targetCol = paste("T", i, sep = "")
    ccm_out = CCM(dataFrame = all_data, E = E, Tp = tp_value,
                  columns = y,
                  target = targetCol,
                  libSizes = libSize,
                  random=T,
                  sample = num_sample,
                  seed=2019)
    col = paste(y, ":", targetCol, sep = "")
    dat=ccm_out %>% select(LibSize,col)
    names(dat)=c("lib","rho")
    test1=mutate(dat,i=i,dis=y,plt=x,E=E,
                 tp_value=tp_value,state=ST)
    rho_surr <- rbind(rho_surr,test1)

  }
  rho_surr
}

plist=list(data=list(df_smapc),
           ST=unique(df_smapc$state),
           y=c('fluP'),
           x=c('o3',"temp","ah"),
           tp_value=-2:0) %>% cross_df()

cl <- makeCluster(cores[1],type = core_type)
```

```
registerDoParallel(cl)
ccm_out=foreach(j = 1:nrow(plist),
                .packages = c("rEDM","tidyverse"),
                .combine=rbind,
                # .export='num_sample',
                .inorder=FALSE)  %dopar% {
                  ccm_out=plist[j,] %>% pmap_df(fn_season_ccm)
                }
stopCluster(cl)

# Calculate the difference in cross-mapping skills obtained by the m
aximum and the minimum library to test convergence property
dat_min <- ccm_out %>% filter(lib<50)
dat_min <- dat_min[order(dat_min$state, dat_min$tp_value, dat_min$pl
t, dat_min$dis, dat_min$i),]
dat_max <- ccm_out %>% filter(lib>50)
dat_max <- dat_max[order(dat_max$state, dat_max$tp_value, dat_max$pl
t, dat_max$dis, dat_max$i),]

dat <- cbind(dat_max,dat_min[,"rho"])
names(dat) <- c("lib","rho_max","i","dis","plt","E","tp_value","ST",
"rho_min")

dat1 <- dat %>% mutate(rho=rho_max-rho_min)
ccm_out <- dat1

ccm_out_raw = ccm_out  %>%
  filter(i==1) %>%
  select(plt, ST,tp_value, rho)

# Calculate the P value of significance test by comparing the origin
al CCM skill against the null distribution of surrogate ones.
ccm_p=ccm_out %>%
  group_by(dis,plt,E,ST,tp_value) %>%
  summarise(p=1-ecdf(rho[i != 1])(rho[i == 1])) %>%
  left_join(ccm_out_raw, by=c("plt", "ST", "tp_value")) %>%
  rename(rho_raw=rho) %>%
  arrange(dis,plt)

ccm_p %>% filter(p==0)
ccm_p %>% filter(rho_raw<=0)

# Adjust extreme P values before meta-significance test
# If P is extremely small approximating 0, then P is deemed as 0.005
 allowing for Fisher's meta-significance test
# If original CCM skill is <0, then P is deemed as 1, that is accept
ing the null hypothesis exactly.

ccm_p=ccm_p %>%
  mutate(p=ifelse(p==0, 0.0005, p)) %>%
  mutate(p=ifelse(rho_raw<=0, 1, p))

# Calculate meta-significance test using Fisher's method.
```

```r
fn_metap=function(var,tp_values,plts,diss){
  df=filter(var,tp_value==tp_values & plt==plts & dis==diss)
  out=allmetap(df$p, method = "sumlog") %>% as.data.frame()
  mutate(out,tp_value=tp_values,plt=plts,dis=diss)
}

plist=list(var=list(ccm_p),
           tp_values=-2:0,
           plts=c("o3","temp","ah"),
           diss=c('fluP')) %>% cross_df()

meta_ccm_p_out=plist %>% pmap_df(fn_metap)

print(meta_ccm_p_out$p, digits=12)

############################### Plotting CCM Causality Test ########
#######################
# Reshape the CCM output: causality test
ccm_causal <- ccm_out %>%
  full_join(ccm_p, by = c("ST","plt","dis",'E','tp_value')) %>%
  mutate(grp=ifelse(i==1,'raw','surr'),
         sig=ifelse(p<0.05 & rho_raw>0, 'sig', 'non_sig'),
         sig=factor(sig, levels=c("non_sig","sig"))) %>%
  group_by(grp,ST,plt,tp_value) %>%
  mutate(Q50=quantile(rho, 0.5),
         Q0=quantile(rho, 0),
         Q95=quantile(rho, 0.95)) %>%
  mutate(Q50=ifelse(grp=='raw',NA,Q50),
         Q0=ifelse(grp=='raw',NA,Q0),
         Q95=ifelse(grp=='raw',NA,Q95))

abc_ST_levels <- str_sort(toupper(unique(ccm_causal$ST)),decreasing=
F)
ccm_causal <- ccm_causal %>%
  mutate(ST=factor(toupper(ST), levels= abc_ST_levels)) %>%
  mutate(plt=factor(plt, levels=c( "o3","ah", "temp"),
                    labels=c("O[3]", "AH", "T")))

# Plot state-specific CCM results with significance test using surro
gate data
mytheme <- theme_bw() +
  theme(panel.border = element_blank(),
        panel.background = element_rect(fill = NA, colour ="grey90"
),
        strip.background = element_rect(fill = NA, colour = NA),
        strip.text.x = element_text(size = 14, color = "gray10",
                                    face='bold',family='serif'),
        panel.grid.major = element_blank(),
        panel.grid.minor = element_blank())+
  theme(legend.position = c(.85, 0.025), legend.box = "horizontal",
        legend.title=element_text(size=18,family='serif'),
        legend.key.width= unit(1.1, 'cm'),
        legend.text = element_text(size=14, color = "black",family='
serif'),
```

```
        legend.spacing.y = unit(0.1, 'cm'),
        legend.background = element_rect(color = NA),
        legend.box.margin = margin(0.1,0.1,0.1,0.1,"cm")) +
  theme(axis.title = element_text(size=20,family='serif'),
        axis.text= element_text(color="black", size=18,family='serif
'),
        axis.line = element_line(size = 0.5, linetype = "solid", col
our = "black"),
        plot.title = element_text(size = 20, hjust=0.5, family='seri
f'))

p_ccm_all_lag1= ccm_causal %>%
  group_by(ST,plt,tp_value) %>%
  spread(grp,rho) %>% filter(tp_value==-1) %>%
  ggplot() +
  geom_errorbar(aes(x=fct_rev(plt),ymin=Q0,ymax=Q95),
                width=0, size=0.8, colour="gray",
                position = position_dodge(0.8)) +
  geom_point(aes(x=fct_rev(plt),y=rho_raw,
                 shape=factor(sig)),
                 size = 4, stroke = 0.5, colour="red",
                 position = position_dodge(0.8)) +
  facet_wrap(~ ST, ncol = 6)+
  scale_shape_manual(name="Surrogate test:", values = c(1,16),
                     labels=c("Non-significant","Significant"),
                     guide=guide_legend(order = 1)) +
  labs(x='', y=expression(paste(Delta, rho["CCM"]))) +
  scale_x_discrete(labels = c(expression("T"), expression("AH"), exp
ression(O[3]))) +
  scale_y_continuous(limits = c(-0.23, 0.51),
                     breaks = c(-0.2, -0.1, 0, 0.1, 0.2, 0.3, 0.4, 0
.5),
                     labels = c('-0.2', '', '0', '', '0.2', '','0.4'
,'')) +
  geom_hline(yintercept = 0, linetype = 2, color = "gray") +
  coord_flip() +
  mytheme

# Plot nation-wide summary of CCM results
dat_label <- tibble(xpos=c('O[3]','AH','T'), ypos= rep(-0.52, 3),
                    lab =c("italic(P[meta]==1.9%*%10^{-7})",
                           "italic(P[meta]==1.4%*%10^{-2})",
                           "italic(P[meta]==1.4%*%10^{-1})"))
p_ccm_vs_1 <- ccm_causal %>%
  filter(grp=="raw" & tp_value==-1) %>%
  ggplot(aes(x=fct_rev(plt), y=rho)) +
  geom_violin(color='gray80') +
  geom_quasirandom(aes(shape=sig), color="red",
                   width=0.25, size=4, alpha=1) +
  scale_shape_manual(values = c(1,16), guide='none')+
  geom_hline(yintercept = 0, linetype = 2, color = "gray") +
  labs(x =  "", y = expression(paste(Delta, rho["CCM"])))+
  scale_x_discrete(labels = c(expression("T"), expression("AH"), exp
ression(O[3]))) +
```

```
  scale_y_continuous(limits = c(-0.6, 0.6),
                     breaks = c(-0.4, -0.3, -0.2, -0.1, 0, 0.1, 0.2,
 0.3, 0.4, 0.5),
                     labels = c('-0.4', '', '-0.2', '', '0', '', '0.
2', '','0.4','')) +
  geom_text(data = dat_label, aes(x=xpos, y=ypos, label=lab), color=
'gray20', parse = TRUE, family='serif', size=5, position = "nudge")
+
  coord_flip()+
  mytheme

p_ccm_vs_1_arrange = ggarrange(NULL,p_ccm_vs_1,NULL,
                               nrow = 1, ncol = 3,
                               widths = c(0.5, 1, 0.5),
                               common.legend = FALSE,
                               align = "hv")

p_ccm_3_all <- ggarrange(p_ccm_all_lag1,
                         p_ccm_vs_1_arrange,
                         nrow = 2, ncol = 1,
                         heights = c(11, 2.5),
                         common.legend = FALSE,
                         align = "hv")

#+ fig.height=12,fig.width=10
p_ccm_3_all
#+
```

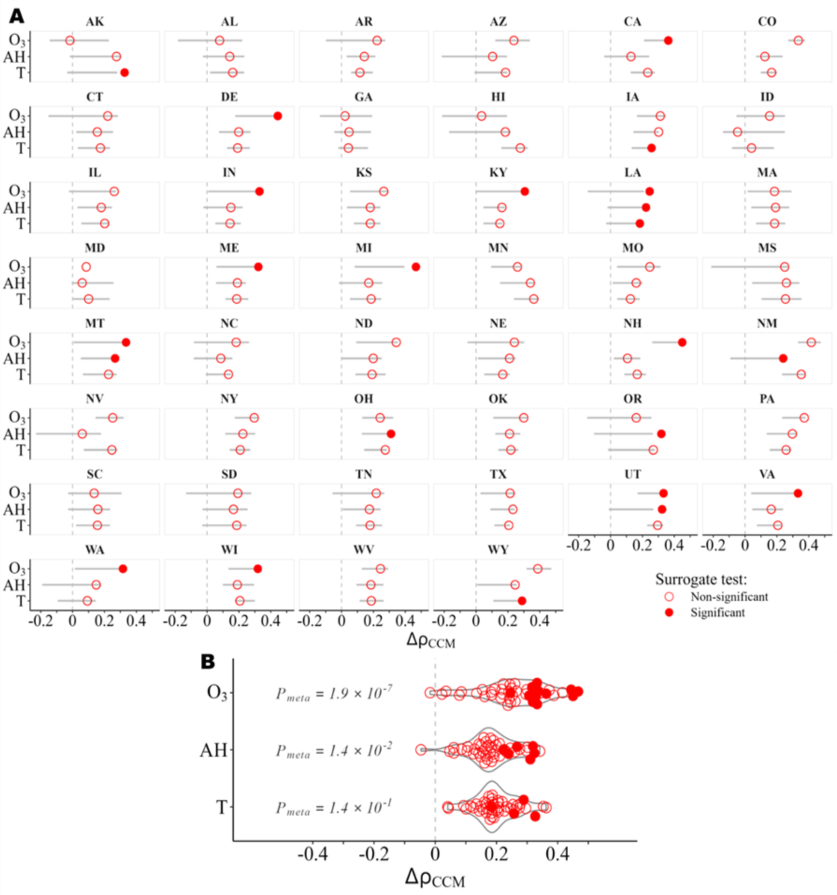

1.4 Effect strength estimation

After CCM causality tests, multivariate *S-map* approach, which is also incorporated within the EDM framework, was employed to probe the direction and magnitude of the influence exerted by potential environmental drivers (such as O_3) on influenza intensity.

```
###### Effect strength
fn_smapc=function(data,ST,plt,dis,tp_value){

  E=E_smapc[E_smapc$ST==ST & E_smapc$dis==dis,"E"]

  df=data %>% filter(state==ST) %>%
    mutate(plt_tp=lag(.data[[plt]],-tp_value)) %>%
    filter(!(is.na(plt_tp))) %>%
    select(date,all_of(dis),plt_tp) %>%
    na.omit()

  M <- nrow(df)

  embed_1=Embed(dataFrame = df, E = E, tau = -1, columns = dis )

  dataFrame = cbind(df[E:M, 'date'],df[E:M, dis],
                    embed_1[E:M, 1:(E-1)], df[E:M, 'plt_tp']  ) %>%
    as.data.frame()

  names(dataFrame)=c('date',dis,letters[1:(E-1)],plt)

  columns = paste(paste(letters[1:(E-1)],collapse =' '), plt ,
                  sep=' ')

  m <- nrow(dataFrame)

  lib=make_pred_nozeroL(dataFrame)
  pred=make_pred_nozeroL(dataFrame)

  theta = best_theta[best_theta$state==ST & best_theta$dis==dis, "th
eta"]

  smap = SMap(dataFrame = dataFrame,
              embedded = TRUE,
              columns = columns,
              target = dis,
              lib = lib,
              pred=pred,
              theta=theta,
              Tp = 1,
              E = E)
  smapc_df=smap$coefficients[c(1,2+E)]

  names(smapc_df)=c('date','effect')
  smapc_df=smapc_df %>%
    mutate(date=lubridate::as_date(date,
                                   origin = lubridate::origin)) %>%
```

```
    mutate(dis=dis,ST=ST, plt=plt,E=E,tp_value=tp_value)
}

plist=list(data=list(df_smapc),
           ST=unique(df_smapc$state),
           dis=c('fluP'),
           plt=c('o3','ah','temp'),
           tp_value=-2:0) %>% cross_df()


cl <- makeCluster(cores[1],type = core_type)
registerDoParallel(cl)
C_out=foreach(i = 1:nrow(plist),
              .packages = c("rEDM","tidyverse","lubridate"),
              .combine=rbind,
              .export='best_theta',
              .inorder=FALSE)  %dopar% {
                C_out=plist[i,] %>% pmap_df(fn_smapc)
              }

stopCluster(cl)

############################### Plotting S-map Effect Size #########
######################
# Reshape the C_out output: effect strength estimates
SEeffect<- C_out %>%
  select(date,ST,tp_value,dis,plt,effect) %>%
  mutate(plt=factor(plt,
                    levels=c("o3","ah", "temp"),
                    labels=c("O[3]","AH", "T"))) %>%
  mutate(tp_value=factor(tp_value,
                         levels=c(0, -1, -2)))

SEeffect_ex <- SEeffect %>% group_by(ST, tp_value,plt,dis) %>%
  filter(effect < quantile(effect, probs=.95, na.rm = T),
         effect > quantile(effect, probs=.05, na.rm = T)) # filter o
ut the extreme values

# Prepare the plotting data set
SE_mean_lag = SEeffect_ex %>%
  filter(dis=="fluP", plt=='O[3]') %>%
  group_by(ST,tp_value) %>%
  dplyr::summarise(median_effect=median(effect,na.rm=TRUE)) %>%
  filter(tp_value==-1) %>%
  select(state=ST, median_effect)

centroid_labels <- usmapdata::centroid_labels("states")
map_labels <- centroid_labels

# Plot state-specific median effect size onto the map
p_o3SE_map <- plot_usmap(data = SE_mean_lag,
                         values = 'median_effect',
                         color = "grey", labels = F, label_color = "
black") +
```

```
  geom_text(data = map_labels,
            ggplot2::aes(x = x, y = y, label = abbr),
            color = "black", family='serif')+
  scale_fill_gradient2(name = expression(paste("EDM effect estimates
: ", O[3] %->% "Flu")),
                       limits = c(-0.31,0.1),
                       breaks = c(-0.3,-0.2,-0.1, 0, 0.1),
                       midpoint = 0,
                       low = "royalblue3", mid = "white", high = "re
d",na.value="gainsboro",
                       guide=guide_colorbar(direction = "horizontal"
,
                                            title.position = "top")
) +
  theme(legend.position=c(0.56,0),
        legend.title=element_text(size=11, family='serif'),
        legend.text = element_text(size=8, color = "black", family='
serif'),
        legend.key.height = unit(0.5, 'cm'),
        legend.key.width = unit(0.8, 'cm'))

print(p_o3SE_map)
```

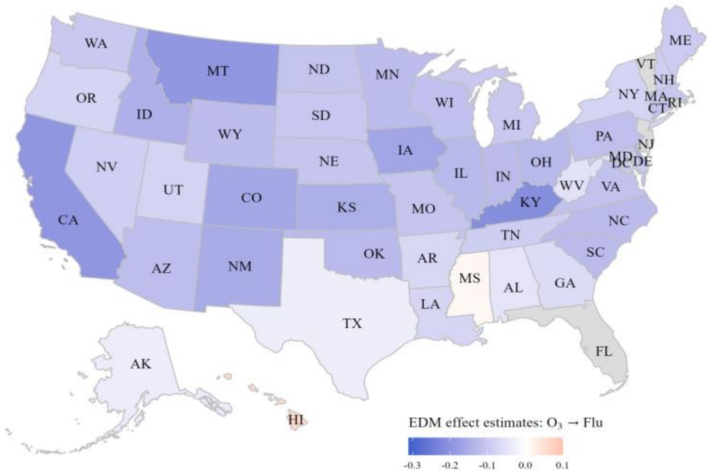

2. PCMCI+

In the PCMCI+ analysis, we also approached the investigation at both the state and national levels. For the national analysis, we utilized a consolidated dataset from 46 states, opting for a direct analysis of merged data rather than conducting a meta-analysis of state-specific results. This is recommended by the developers of PCMCI+. To ensure the validity of our time series analysis, we strategically inserted rows containing 'NA' values between strata representing different flu seasons. This step was crucial to prevent any potential mixing of data from different seasons. Additionally, we normalized the data from each state before amalgamating them, ensuring a coherent magnitude for the subsequent national-level analysis. The codes for these data processing is saved under the Github file of `"PCMCI_data_processing.R"`. Then the data processed by R software is passed to Python environment for downstream PCMCI+ analysis.

2.1 Prepare data and functions

Initially, we import the required libraries and set the working directory. Following this, we define several functions tailored for the PCMCI+ analysis:

- `get_data`: This function converts our imported data set into a format suitable for the PCMCI+ algorithm. Specifically, it extracts data for a designated state and the selected variables, returning the processed data as a numpy array.

```
# Import necessary libraries
import os
import pandas as pd
from matplotlib import pyplot as plt
from tigramite import data_processing as pp
from tigramite import plotting as tp
from tigramite.pcmci import PCMCI
from tigramite.independence_tests import ParCorr
import numpy as np

# Setting the directory
os.chdir("//Mac/Home/Desktop/PRJ/USA_FLU")

# Function for preparing the values of the variables specified by 'v
ar_names'
# in the state specified by 'state' as numpy array of shape (T, N),
where
# T is the number of time steps and N the number of variables
def get_data(data_all, state, var_names=['Flu', '$\mathregular{O_3}$
', 'T', 'AH']):

    # Select the state
    if state == "Overall":
        data_out = data_all
    else:
        data_out = data_all.loc[data_all[r'state'] == state]

    # Select the columns
    data_out = data_out[var_names]

    # Turn into numpy array
    data_out = data_out.values

    # Return
    return data_out
```

2.2 Link selections

- `get_selected_links`: This function is for including prior knowledge. It is designed to establish potential relationships or links between the different variables based on their time lags. The purpose is to give prior information about which variable might influence or be influenced by other variables at different time lags. The outcome of this function is a dictionary (`selected_links`) where keys are indices representing the variables and values are lists of tuples. Each tuple contains the index of a potentially

influencing variable and the time lag. By this function, we can include prior knowledge into our PCMCI+ analyses. This is a crucial step to guide the PCMCI+ in understanding potential causal relationships.

```
def get_selected_links(var_names, tau_min, tau_max):

    # Get index of the temperature variable, if it exists
    if 'T' in var_names:
        temp_idx = np.argwhere(np.array(var_names) == 'T')[0, 0]
    else:
        temp_idx = None

    # Get index of the humidity variable, if it exists
    if 'AH' in var_names:
        humid_idx = np.argwhere(np.array(var_names) == 'AH')[0, 0]
    else:
        humid_idx = None

    # Build dictionary
    selected_links = {}

    for idx, var in enumerate(var_names):

        if var == 'Flu':
            # Flu may be influenced by all variables at all lags
            selected_links[idx] = [(other_idx, -tau) for other_idx,
other_var in enumerate(var_names)
                                               for tau in range(tau_min, tau_max
 + 1)]
        elif var == 'AH':
            # Humiditiy may be influenced by itself at all lags
            selected_links[idx] = [(idx, -tau) for tau in range(tau_
min, tau_max + 1)]

            # Humidity may also be influenced by temperature at non-
zero lags
            selected_links[idx] = [(temp_idx, -tau) for tau in range
(max(1, tau_min), tau_max + 1)]

        elif var == 'T':
            # Temperature may be influenced by itself at all lags
            selected_links[idx] = [(idx, -tau) for tau in range(tau_
min, tau_max + 1)]

            # Temperature may also be influenced by humidity at non-
zero lags
            selected_links[idx] = [(humid_idx, -tau) for tau in rang
e(max(1, tau_min), tau_max + 1)]

        else:
            # All other variables, here this is O3, may be influence
d by all variables other than Flu
            selected_links[idx] = [(other_idx, -tau) for other_idx,
other_var in enumerate(var_names)
```

```
                                    for tau in range(tau_min, tau_max
 + 1) if
                                    ((other_var != 'Flu')]
    # Return
    return selected_links
```

2.3 PCMCI+ algorithms

- `apply_pcmci`: This function serves as the central engine for executing the PCMCI+ algorithms on the data set. It systematically prepares, configures, and executes the PCMCI+ analysis and presents the results graphically. The function displays a graphical representation of the PCMCI+ results, highlighting causal relationships. Additionally, it returns the results object which contains detailed statistics and information about the analysis.

```
def apply_pcmci(data_all,
                state,
                var_names,
                tau_min,
                tau_max,
                pc_alpha,
                verbosity):
    # Get the data and mask
    data, mask = get_data (data_all=data_all,
                           state=state,
                                                                   var_na
mes=var_names)

    # Prepare the DataFrame object
    dataframe = pp.DataFrame(data,
                             var_names=var_names,
                             missing_flag=999.)

    # Prepare the independence test and PCMCI object
    parcorr = ParCorr()
    pcmci = PCMCI(dataframe=dataframe,
                  cond_ind_test=parcorr,
                  verbosity=verbosity)

    # Get the selected_links arguement
    selected_links = get_selected_links(var_names,
                                        tau_min,
                                        tau_max)

    # Run PCMCI^+ with these parameters
    results = pcmci.run_pcmciplus(tau_min=tau_min,
                                  tau_max=tau_max,
                                  pc_alpha=pc_alpha,
                                  selected_links=selected_links)

    tp.plot_graph(
        arrow_linewidth=5.0,
        figsize=(12*0.4, 5*0.4),
        vmin_edges=-0.5,
```

```
        vmax_edges=0.5,
        node_label_size=9,
        node_size=0.5,3
        link_label_fontsize=5,
        val_matrix=results['val_matrix'],
        graph=results['graph'],
        var_names=var_names,
        link_colorbar_label='cross-MCI (edges)',
        node_colorbar_label='auto-MCI (nodes)',
        label_fontsize=8,
        network_lower_bound=0.2,
        show_colorbar=0
    );

    plt.show()
```

Below are national-wise and state-wise results. Directional curved and straight edges between nodes represent the lagged and contemporaneous causal dependencies, respectively. A lagged link is labelled by the lag number on the curve. Node colour denotes autocorrelation strength (i.e., auto-MCI [Momentary Conditional Independence] value); edge colour depicts the causal strength (i.e., cross-MCI) estimated via partial correlation.

```
data_all = pd.read_csv("usaflu_norm.csv")
data_all.rename(columns={'fluP': 'Flu',
                         'o3': '$\mathregular{O_3}$',
                         'temp': 'T',
                         'ah': 'AH'}, inplace=True)

pc_alpha = 0.001

results = apply_pcmci(data_all=data_all,

                      state="Overall",
                      var_names=['Flu', '$\mathregular{O_3}$', 'T',
'AH'],
                      tau_min=0,
                      tau_max=2,
                      pc_alpha=pc_alpha,
                      verbosity=0,
                      name="Overall"
                      )

data_all = pd.read_csv("usaflu_raw.csv")
data_all.rename(columns={'fluP': 'Flu',
                         'o3': '$\mathregular{O_3}$',
                         'temp': 'T',
                         'ah': 'AH'}, inplace=True)


states = ["AK", "AL", "AR", "AZ", "CA", "CO", "CT", "DE", "GA", "HI"
, "IA", "ID", "IL", "IN", "KS",
"KY", "LA", "MA", "MD", "ME", "MI", "MN", "MO", "MS", "MT", "NC", "N
D", "NE", "NH", "NM",
```

```
"NV", "NY", "OH", "OK", "OR", "PA", "SC", "SD", "TN", "TX", "UT", "V
A", "WA", "WI", "WV",
"WY" ]

pc_alpha = 0.05
for state in states:
    results = apply_pcmci(data_all=data_all,
                          state=state,
                          var_names=['Flu', '$\mathregular{O_3}$', 'T',
'AH'],
                          tau_min=0,
                          tau_max=2,
                          pc_alpha=pc_alpha,
                          verbosity=0
                            )
```

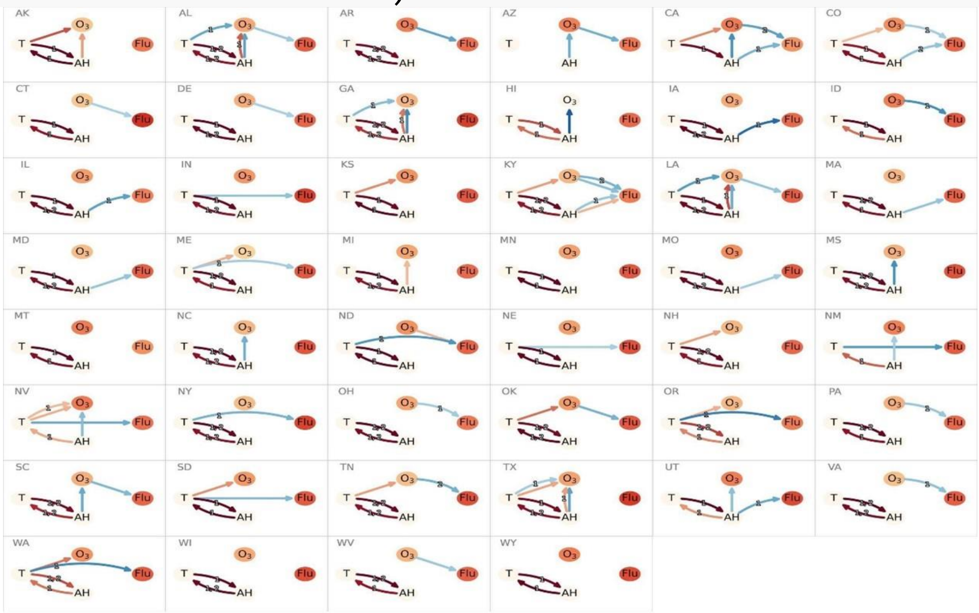

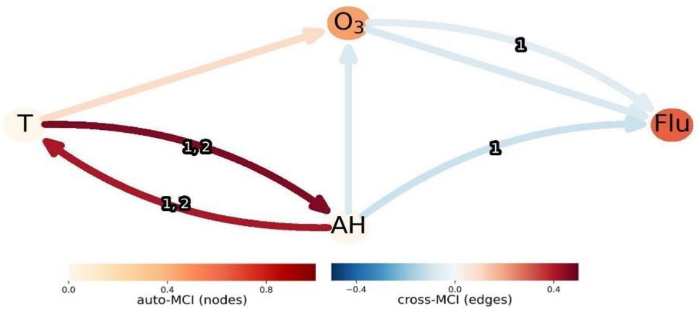

3. GLM

Finally, GLM regression is used to assess the relationship of O_3, AH, and T with influenza intensity in each state after proper adjustment for covariates.

```
###### Load packages & data
packages=c('tidyverse', 'lubridate', 'dlnm', 'splines', 'tsModel', '
gnm', 'ggpubr', 'metafor', 'mgcv', 'imputeTS')
lapply(packages, require, character.only=T)

load("Data_fluseason.rda")
```

```
df <- usa_Flu_P_proxy_Data_B

dfA <- df %>%
  mutate(time = as.numeric(date),
         year = year(date),
         month = month(date))
```

3.1 State-specific regression

Given that influenza intensity is represented by proportional data, we utilized a quasi-binomial link with a logit function (i.e., $log[Y/(1-Y)]$). To address the possible confounding influences of unaccounted factors, we incorporated year-specific dummy variables to recognize long-term trends, and month-specific dummy variables to account for seasonal variations in the model. To compensate for the significant autocorrelation due to disease spread, we included the logarithm of the outcome variable from the previous week (i.e., $log(Y_{t-1})$) as an additional covariate in our model. In the GLM analysis, we carried out mutual adjustments to isolate the potential unique effect of each environmental factor on influenza intensity. For instance, when evaluating the association between O_3 and influenza, the current-week AH and T were also incorporated into the model as linear terms to account for their potential impacts.

```
###### State-level GLM
gam_function <- function(dat, Y='fluP', x1='value', xlag=0, cityname
, variable) {

  df <- dat %>%
    filter(state == cityname) %>%
    mutate(
      loglag1 = log(Lag(fluP, 1)),
      loglag2 = Lag(loglag1, 1)
    )

  formula_str <- switch(variable,
                        o3   = paste(Y, '~Lag(', x1, ',', xlag, ')+
Lag(ah,', xlag, ')+ Lag(temp,', xlag, ')+ as.factor(year) + as.facto
r(month) + loglag1', sep=''),
                        ah   = paste(Y, '~Lag(', x1, ',', xlag, ')+
Lag(o3,', xlag, ')+ Lag(temp,', xlag, ')+ as.factor(year) + as.facto
r(month) + loglag1', sep=''),
                        temp = paste(Y, '~Lag(', x1, ',', xlag, ')+
Lag(o3,', xlag, ')+ Lag(ah,', xlag, ')+ as.factor(year) + as.factor(
month) + loglag1', sep='')
  )

  fit <- gam(as.formula(formula_str), data=df, family=quasibinomial,
 na.action=na.omit)
  summ <- summary(fit)

  outA <- data.frame(
    beta = summ$p.coeff[2],
    se   = summ$se[2],
    t    = summ$p.t[2],
    p    = summ$p.pv[2],
```

```
    state = cityname,
    lag   = xlag
  )

  return(outA)
}

variables <- c("o3", "ah", "temp")
results <- list()

for (var in variables) {
  plist <- list(
    dat      = list(dfA),
    Y        = 'fluP',
    x1       = var,
    xlag     = 0:2,
    cityname = unique(dfA$state),
    variable = var
  ) %>% cross_df()

  results[[var]] <- plist %>% pmap_df(gam_function)
}

# Store the results for easy access
out_o3   <- results$o3
out_ah   <- results$ah
out_temp <- results$temp
```

3.2 Random-effects meta-analysis

Then, the state-wise and lag-specific effect estimates were pooled with random-effects meta-analysis (using restricted maximum-likelihood estimator for the between-study variance) models. Note that, the state-level and nation-wide significance threshold were set at 0.05 and 0.001, respectively, being consistent with CCM and PCMCI+. In the context of GLM, the 95% (99.9%) confidence intervals of regression coefficients (β) were computed as appropriate.

```
###### Meta analysis of GLM beta results
fmeta=function(dat){
  out=dat

  GAMresCI  =  out %>%
    mutate(betalow=beta-1.96*se,
           betahigh=beta+1.96*se) %>% # 95% CI
    mutate(state=as.factor(state),
           lag=factor(lag, levels = c(0,1,2),
                      labels = c("Lag 0","Lag 1","Lag 2"))) %>%
    select(state,lag,beta,betalow,betahigh,p)

  meta.ci.result  =  as.data.frame(matrix(rep(NA,18),nrow=3))
  names(meta.ci.result)  =  names(GAMresCI)

  for (i in 1:3) {
    out.lag  =  out %>%
```

```
      filter(lag==i-1)
    meta  =  rma(yi=beta, sei=se, slab=state, method="REML", data=ou
t.lag, level=99.9)
    meta.re  =  with(meta, c(b, ci.lb, ci.ub,pval))
    meta.ci.result[i,1] = "All"
    meta.ci.result[i,2] = paste('Lag',i-1)
    meta.ci.result[i,3] = meta.re[1]
    meta.ci.result[i,4] = meta.re[2]
    meta.ci.result[i,5] = meta.re[3]
    meta.ci.result[i,6] = meta.re[4]
  }

  meta.ci.result  =  meta.ci.result %>% as.data.frame()

  abc_levels=str_sort(toupper(unique(GAMresCI$state)),decreasing=T)

  GAMresCI_all  =  rbind(GAMresCI, meta.ci.result) %>%
    mutate(type=factor(ifelse(state=="All",2,1))) %>%
    mutate(state=factor(toupper(state),
                        levels=c(abc_levels,"ALL"))) %>%
    as.tibble()
}

gam_o3=fmeta(out_o3)
gam_ah=fmeta(out_ah)
gam_temp=fmeta(out_temp)
```

To quantify the effect size, the regression coefficients are scaled by the SD of each predictor. As a result, the effects from the GLM can be understood as the alteration in the logit of influenza intensity for every SD increment in the environmental factors, such as O_3.

```
# Calculate overall and state-specific SD of each environmental pred
ictor
df_SD_all=dfA %>%
  dplyr::summarize(o3=sd(o3), ah=sd(ah), temp=sd(temp)) %>%
  gather(plt, SD) %>%
  mutate(state='ALL')

df_SD_ST <- dfA %>% group_by(state) %>%
  dplyr::summarize(o3=sd(o3), ah=sd(ah), temp=sd(temp)) %>%
  gather(plt, SD, -state) %>%
  mutate(state=toupper(state))

df_SD <- rbind(df_SD_ST, df_SD_all%>%select(state,plt,SD))

# Transform beta to effect size of each SD change
df1=gam_o3  %>% as.data.frame() %>% mutate(plt='o3')
df2=gam_ah  %>% as.data.frame() %>% mutate(plt='ah')
df3=gam_temp %>% as.data.frame() %>% mutate(plt='temp')

df_gam_final=rbind(df1,df2,df3) %>%
  left_join(df_SD,by=c("state",'plt')) %>%
  mutate(Size=beta*SD,SizeL=betalow*SD,SizeH=betahigh*SD) %>%
```

```
  select(state, plt, lag, Size, SizeL, SizeH, gam.p=p) %>%
  mutate(sig=ifelse(gam.p<0.05, 'sig', 'non_sig'),
         sig=factor(sig, levels=c("non_sig","sig")),
         plt=factor(plt, levels=c('temp', 'ah', 'o3')))

###################### Plot ######################
mytheme <- theme_bw() +
  theme(panel.border = element_blank(),
        panel.background = element_rect(fill = NA, colour ="grey90"
),
        strip.background = element_rect(fill = NA, colour = NA),
        strip.text.x = element_text(size = 14, color = "gray10",
                                    face='bold',family='serif'),
        panel.grid.major = element_blank(),
        panel.grid.minor = element_blank(),
        axis.line = element_line(size = 0.5, linetype = "solid",
                                 colour = "black"))+
  theme(legend.position = c(.85, 0.025), legend.box = "horizontal",
        legend.title=element_text(size=18,family='serif'),
        legend.key.width= unit(1.1, 'cm'),
        legend.text = element_text(size=14, color = "black",family='
serif'),
        legend.spacing.y = unit(0.1, 'cm'),
        legend.background = element_rect(color = NA),
        legend.box.margin = margin(0.1,0.1,0.1,0.1,"cm")) +
  theme(axis.title = element_text(size=20,family='serif'),
        axis.text= element_text(color="black", size=18,family='serif
'),
        plot.title = element_text(size = 20, hjust=0.5, family='seri
f'))

# Plotting tate-specific GLM results
abc_levels=str_sort(unique(df_gam_final$state)[1:46],decreasing=F)

dat_glm_ST <- df_gam_final %>%
  filter(state!='ALL' & lag=='Lag 1') %>%
  mutate(state=factor(state, levels= abc_levels))

p_glm_ST_lag1= ggplot(dat_glm_ST) +
  geom_errorbar(aes(ymin=SizeL, ymax=SizeH, x=plt), color='gray',
                position = position_dodge(0.8), width=0, size=0.8) +
  geom_point(aes(y=Size, x=plt, shape=sig),
             color='red', size=4, stroke = 0.5,
             position = position_dodge(0.8))+
  facet_wrap(~ state, ncol = 6)+
  scale_shape_manual(name="Statistical significance test:", values =
 c(1,16),
                     labels=c("Non-significant","Significant")) +
  labs(x='',y=expression(paste(beta, ' estimates in GLM'))) +
  scale_x_discrete(labels = c(expression("T"), expression("AH"), exp
ression(O[3]))) +
  geom_hline(yintercept = 0, linetype = 2, color = "grey") +
  coord_flip() +
  mytheme
```

```
# Plotting meta-analyzed GLM results
dat_glm_ALL <- df_gam_final %>%
  filter(state=='ALL' & lag=='Lag 1')

p_glm_vs_1 <- ggplot(dat_glm_ALL) +
  geom_errorbar(aes(ymin=SizeL, ymax=SizeH, x=plt), color='gray',
                position = position_dodge(0.8), width=0,linewidth=1)
 +
  geom_point(aes(y=Size, x=plt, shape=sig),
             color='red', size=4, stroke = 0.5,
             position = position_dodge(0.8))+
  scale_shape_manual(guide="none",values = c(1,16)) +
  labs(x='',y=expression(paste(beta, ' estimates in GLM'))) +
  scale_x_discrete(labels = c(expression("T"), expression("AH"), exp
ression(O[3]))) +
  geom_hline(yintercept = 0, linetype = 2, color = "grey") +
  coord_flip() +
  mytheme

p_glm_vs_1_arrange = ggarrange(NULL,p_glm_vs_1,NULL,
                               nrow = 1, ncol = 3,
                               widths = c(0.5, 1, 0.5),
                               common.legend = FALSE,
                               align = "hv")

p_glm_3_all <- ggarrange(p_glm_ST_lag1,
                         p_glm_vs_1_arrange,
                         nrow = 2, ncol = 1,
                         heights = c(11, 2.5),
                         common.legend = FALSE,
                         align = "hv")

#+ fig.height=12,fig.width=10
p_glm_3_all
#+
```

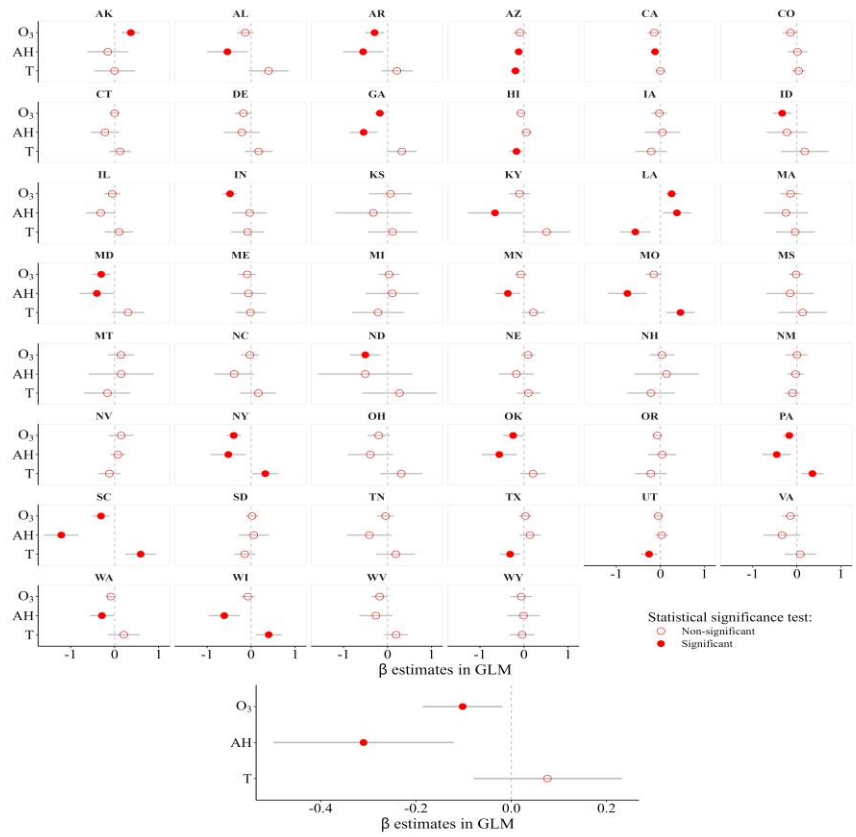
AK
AL
AR
AZ
CA
CO
CT
DE
GA
HI
IA
ID
IL
IN
KS
KY
LA
MA
MD
ME
MI
MN
MO
MS
MT
NC
ND
NE
NH
NM
NV
NY
OH
OK
OR
PA
SC
SD
TN
TX
UT
VA
WA
WI
WV
WY
O_3
AH
T
-1
0
1
β estimates in GLM
Statistical significance test:
Non-significant
Significant
-0.4
-0.2
0.0
0.2
β estimates in GLM

References

[1] Sugihara, George, Robert May, Hao Ye, Chih-Hao Hsieh, Ethan Deyle, Michael Fogarty, and Stephan Munch. 2012. "Detecting Causality in Complex Ecosystems." *Science* 338 (6106): 496–500.

[2] Runge, Jakob, Peer Nowack, Marlene Kretschmer, Seth Flaxman, and Dino Sejdinovic. 2019. "Detecting and Quantifying Causal Associations in Large Nonlinear Time Series Datasets." *Sci Adv* 5 (11): eaau4996.

[3] Runge, Jakob. 2020. "Discovering Contemporaneous and Lagged Causal Relations in Autocorrelated Nonlinear Time Series Datasets." In *Proceedings of the 36th Conference on Uncertainty in Artificial Intelligence (UAI)*, edited by Jonas Peters and David Sontag, 124:1388–97. Proceedings of Machine Learning Research. PMLR.

[4] Bhaskaran, Krishnan, Antonio Gasparrini, Shakoor Hajat, Liam Smeeth, and Ben Armstrong. 2013. "Time Series Regression Studies in Environmental Epidemiology." *International Journal of Epidemiology* 42 (4): 1187–95.

[5] Cheng, B., and H. Tong. 1992. "On Consistent Nonparametric Order Determination and Chaos." *Journal of the Royal Statistical Society* 54 (2): 427–49.

[6] Deyle, Ethan R., Michael Fogarty, Chih-Hao Hsieh, Les Kaufman, Alec D. MacCall, Stephan B. Munch, Charles T. Perretti, Hao Ye, and George Sugihara. 2013. "Predicting Climate Effects on Pacific Sardine." *Proceedings of the National Academy of Sciences of the United States of America* 110 (16): 6430–35.

[7] Deyle, Ethan R., M. Cyrus Maher, Ryan D. Hernandez, Sanjay Basu, and George Sugihara. 2016. "Global Environmental Drivers of Influenza." *Proceedings of the National Academy of Sciences of the United States of America* 113 (46): 13081–86.

[8] Ye, Hao, Richard J. Beamish, Sarah M. Glaser, Sue C. H. Grant, Chih-Hao Hsieh, Laura J. Richards, Jon T. Schnute, and George Sugihara. 2015. "Equation-Free Mechanistic Ecosystem Forecasting Using Empirical Dynamic Modeling." *Proceedings of the National Academy of Sciences of the United States of America* 112 (13): E1569–76.

[9] Angrist, Joshua D., and Jörn-Steffen Pischke. 2009. *Mostly Harmless Econometrics: An Empiricist's Companion*. Princeton University Press.

[10] Gaure, Simen. 2013. "Lfe: Linear Group Fixed Effects." *The R Journal* 5 (2): 104–17.
