# Supplementary material for: Ozone as an environmental driver of influenza
Source: Nat Commun. 2024 May 4;15:3763. doi: 10.1038/s41467-024-48199-z (PMC11069565; doi:10.1038/s41467-024-48199-z)
Supplement: Supplementary file 3 — Reporting Summary [file 41467_2024_48199_MOESM3_ESM.pdf]

nature portfolio

Corresponding author(s): Linwei Tian

Last updated by author(s): Apr 1, 2024

Reporting Summary

Nature Portfolio wishes to improve the reproducibility of the work that we publish. This form provides structure for consistency and transparency in reporting. For further information on Nature Portfolio policies, see our Editorial Policies and the Editorial Policy Checklist.

Statistics

For all statistical analyses, confirm that the following items are present in the figure legend, table legend, main text, or Methods section.

n/a	Confirmed	
☐	☒	The exact sample size (n) for each experimental group/condition, given as a discrete number and unit of measurement
☒	☐	A statement on whether measurements were taken from distinct samples or whether the same sample was measured repeatedly
☐	☒	The statistical test(s) used AND whether they are one- or two-sided<br>*Only common tests should be described solely by name; describe more complex techniques in the Methods section.*
☐	☒	A description of all covariates tested
☐	☒	A description of any assumptions or corrections, such as tests of normality and adjustment for multiple comparisons
☐	☒	A full description of the statistical parameters including central tendency (e.g. means) or other basic estimates (e.g. regression coefficient) AND variation (e.g. standard deviation) or associated estimates of uncertainty (e.g. confidence intervals)
☐	☒	For null hypothesis testing, the test statistic (e.g. F, t, r) with confidence intervals, effect sizes, degrees of freedom and P value noted<br>*Give P values as exact values whenever suitable.*
☒	☐	For Bayesian analysis, information on the choice of priors and Markov chain Monte Carlo settings
☐	☒	For hierarchical and complex designs, identification of the appropriate level for tests and full reporting of outcomes
☐	☒	Estimates of effect sizes (e.g. Cohen's d, Pearson's r), indicating how they were calculated

Our web collection on statistics for biologists contains articles on many of the points above.

Software and code

Policy information about availability of computer code

Data collection	No software was used for data collection.
Data analysis	Our analyses were conducted in the R (version 4.1.1) and Python (version 3.8). The “rEDM” package (version 1.9.2), a collection of methods for Empirical Dynamic Modeling (EDM), was utilized to generate the Convergent Cross Mapping (CCM) and Sequential locally weighted global linear map (S-map) results. The “Tigramite” package (version 4.2) was harnessed to complete the Peter-Clark-momentary-conditional-independence plus (PCMCI+) graphical modelling. The “mgcv” (version 1.8-37) was adopted to fit the quasi-Binomial regression model.

For manuscripts utilizing custom algorithms or software that are central to the research but not yet described in published literature, software must be made available to editors and reviewers. We strongly encourage code deposition in a community repository (e.g. GitHub). See the Nature Portfolio guidelines for submitting code & software for further information.

Data

Policy information about availability of data

All manuscripts must include a data availability statement. This statement should provide the following information, where applicable:

- Accession codes, unique identifiers, or web links for publicly available datasets
- A description of any restrictions on data availability
- For clinical datasets or third party data, please ensure that the statement adheres to our policy

The raw data on influenza and influenza-like illness are publicly available at https://www.cdc.gov/flu/weekly/index.htm. The ozone data used in this study are

publicly available at http://www.igacproject.org/activities/TOAR. The climate data used in this study are publicly available at https://www.ncdc.noaa.gov/data-access/land-based-station-data/land-based-datasets. The data set supporting the findings of this work is available at the GitHub repository: https://zenodo.org/records/10892898.

Research involving human participants, their data, or biological material

Policy information about studies with human participants or human data. See also policy information about sex, gender (identity/presentation), and sexual orientation and race, ethnicity and racism.

Reporting on sex and gender	N/A
Reporting on race, ethnicity, or other socially relevant groupings	N/A
Population characteristics	N/A
Recruitment	N/A
Ethics oversight	N/A

Note that full information on the approval of the study protocol must also be provided in the manuscript.

Field-specific reporting

Please select the one below that is the best fit for your research. If you are not sure, read the appropriate sections before making your selection.

☐ Life sciences ☐ Behavioural & social sciences ☒ Ecological, evolutionary & environmental sciences

For a reference copy of the document with all sections, see nature.com/documents/nr-reporting-summary-flat.pdf

Ecological, evolutionary & environmental sciences study design

All studies must disclose on these points even when the disclosure is negative.

Study description	This study applied 3 distinct methods, i.e., Convergent Cross Mapping (CCM) which is based on state-space reconstruction theory, a graphical modelling approach called Peter-Clark-momentary-conditional-independence plus (PCMCI+), and a regression method Generalized Linear Model (GLM) to explore the potential environmental divers (ozone, absolute humidity, and air temperature) of influenza activity among the general population, using state-level surveillance time series data (during 2010-2015; 173 weeks) in the USA.
Research sample	State-level weekly data on laboratory-confirmed influenza and medical visits for influenza-like illness (ILI) were retrieved from the USA Center for Disease Control and Prevention (CDC) website.<br>Ambient ozone data in the USA were retrieved from the Tropospheric Ozone Assessment Report (TOAR) of the International Global Atmospheric Chemistry (IGAC); station-based daily maximum 8-hour average ozone levels (ppb) were extracted and averaged by state for analysis.<br>State-level weather data were retrieved from the National Center for Environmental Information (NCEI), the National Oceanic and Atmospheric Administration (NOAA); daily averages of air temperature (?Fahrenheit) and dew point temperature (Fahrenheit), computed from hourly land-based station observations, were extracted and converted to Celsius values. Daily absolute humidity was then calculated from the dew point and air temperature, following standard meteorological formulas.<br>The rationale for sampling timespan is that the state-level influenza and ILI data online had not consistent format over the past years; the only available time period of a consistent variable and data format for all states in the USA was 2010-2015. The samples represent the aggregate exposure levels to environmental factors (ozone, absolute humidity, and air temperature) and influenza activity among the general population of the USA during 2010-2015.
Sampling strategy	The final dataset included 173 weeks of measurements on environmental variables and influenza activity at the state level in the USA. This sampling strategy was based on the data availability from each source.
Data collection	The raw data on influenza and influenza-like illness were recorded by the USA Center for Disease Control and Prevention (CDC); the ozone data were recorded by Tropospheric Ozone Assessment Report (TOAR) of the International Global Atmospheric Chemistry (IGAC); the weather data were recorded by National Center for Environmental Information (NCEI), the National Oceanic and Atmospheric Administration (NOAA).
Timing and spatial scale	Timing: from October 3, 2010 to May 31, 2015 (The rationale is that the state-level influenza and influenza-like illness data online had not consistent format over the past years; the only available time period of a consistent variable and data format for all states in the USA was 2010-2015);<br>Frequency: weekly (Only weekly influenza data are available from the data source);<br>Periodicity: from October through May per year, excluding months from June to September (non-influenza season) which contain little causal information for exploration;<br>Spatial: state by state, the USA.

Data exclusions	This study only focused on the influenza season (that is considered October through May) in the USA for analysis. States which have at least 3 consecutive years of available influenza data are included. Out of the 50 states, 46 were finally eligible for study; states of Vermont, Rhode Island, New Jersey, and Florida were excluded.
Reproducibility	For random number generation, we used a fixed seed '2019' to enable reproducible runs with randomly generated libraries in CCM analysis, and randomly generated surrogate data for statistical significance tests. Three divergent methods simultaneously produced results revealing a negative impact of ozone on influenza intensity.
Randomization	Real-world observational data were contrasted with randomly generated seasonal surrogate data for statistical significance test in the CCM analysis.
Blinding	Blinding is not relevant to this study, since no group allocation or intervention is conducted in this observational study using aggregate time series data.

Did the study involve field work? ☐ Yes ☒ No

Reporting for specific materials, systems and methods

We require information from authors about some types of materials, experimental systems and methods used in many studies. Here, indicate whether each material, system or method listed is relevant to your study. If you are not sure if a list item applies to your research, read the appropriate section before selecting a response.

Materials & experimental systems

n/a	Involved in the study
☒	☐ Antibodies
☒	☐ Eukaryotic cell lines
☒	☐ Palaeontology and archaeology
☒	☐ Animals and other organisms
☒	☐ Clinical data
☒	☐ Dual use research of concern
☒	☐ Plants

Methods

n/a	Involved in the study
☒	☐ ChIP-seq
☒	☐ Flow cytometry
☒	☐ MRI-based neuroimaging

Plants

Seed stocks	*Report on the source of all seed stocks or other plant material used. If applicable, state the seed stock centre and catalogue number. If plant specimens were collected from the field, describe the collection location, date and sampling procedures.*
Novel plant genotypes	*Describe the methods by which all novel plant genotypes were produced. This includes those generated by transgenic approaches, gene editing, chemical/radiation-based mutagenesis and hybridization. For transgenic lines, describe the transformation method, the number of independent lines analyzed and the generation upon which experiments were performed. For gene-edited lines, describe the editor used, the endogenous sequence targeted for editing, the targeting guide RNA sequence (if applicable) and how the editor was applied.*
Authentication	*Describe any authentication procedures for each seed stock used or novel genotype generated. Describe any experiments used to assess the effect of a mutation and, where applicable, how potential secondary effects (e.g. second site T-DNA insertions, mosiacism, off-target gene editing) were examined.*
